# Supplementary material for: T-square resistivity without Umklapp scattering in dilute metallic Bi2O2Se
Source: Nat Commun. 2020 Jul 31;11:3846. doi: 10.1038/s41467-020-17692-6 (PMC7395108; doi:10.1038/s41467-020-17692-6)
Supplement: Supplementary file 1 — Supplementary Information [file 41467_2020_17692_MOESM1_ESM.pdf]

Supplementary information

T-square resistivity without Umklapp scattering in dilute metallic Bi_2O_2Se

Wang et al.

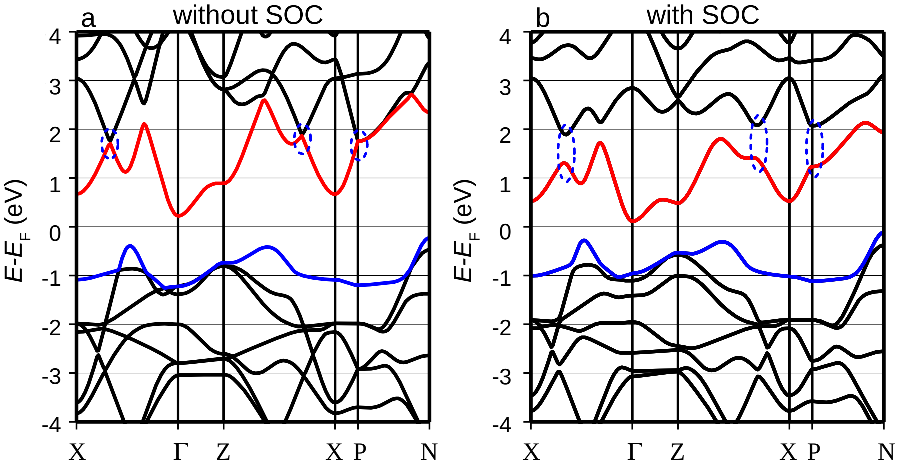

Supplementary Figure 1: **Electronic structure from DFT calculations for Bi_2O_2Se.** **a**: without spin-orbit coupling (SOC). **b** with spin-orbit coupling. The red and blue bands are the lowest conducting band and highest valence band respectively. SOC has little effect on the regime of interests close to the conducting band minimum, but strongly splits conducting bands at higher energy levels as marked by the dashed ellipses.

Supplementary Table 1: **Comparison of crystal and band structure between Bi_2O_2Se and $SrTiO_3$.** The only similarity between Bi_2O_2Se and $SrTiO_3$ is that they both host a single tiny Fermi pocket at Γ point. These observations indicate that the special case of T-square resistivity in absence of inter-band and Umklapp scattering is non-unique and can be observed in different systems with distinct crystal and band structures.

	Bi_2O_2Se	$SrTiO_3$
Crystal structure	layered tetragonal<br>Anti-$ThCr_2Si_2$<br>I4/mmm	3D cubic<br>Perovskite<br>Pm-3m
Band position	Γ point	Γ point
Band dispersion	parabolic	non-parabolic
Fermi pocket	ellipsoid	squeezed ellipsoid

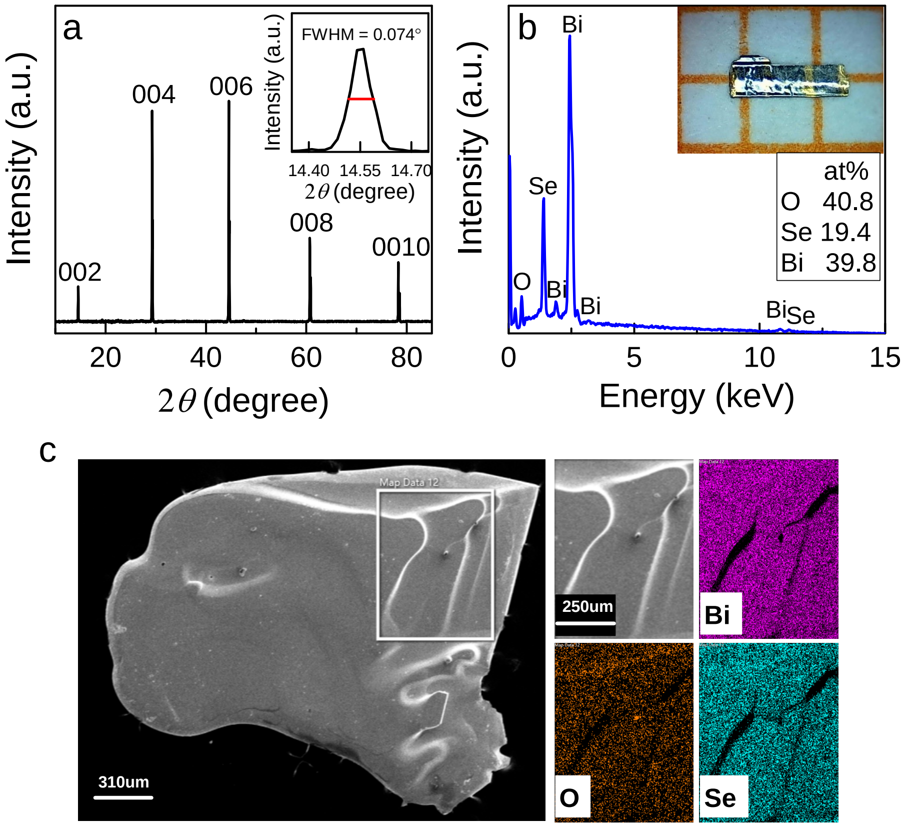

Supplementary Figure 2: **Characterization of Bi_2O_2Se.** **a** The (00l) X-ray diffraction pattern of a single crystal. It indicates that the cleavage plane is along ab-plane. The inset is the zoom-in pattern of the (002) peak, whose full width at half maximum (FWHM) amounts to 0.074° implying good crystallization. **b** The EDX spectrum of Bi_2O_2Se. The molar ratio between Bi, O and Se is very close to the stoichiometric value. The inset is a photograph of one specimen with millimeter-size, on which gold stripes were evaporated to achieve ohmic contacts. **c** SEM images and the element mapping data. It shows that each element is uniformly distributed.

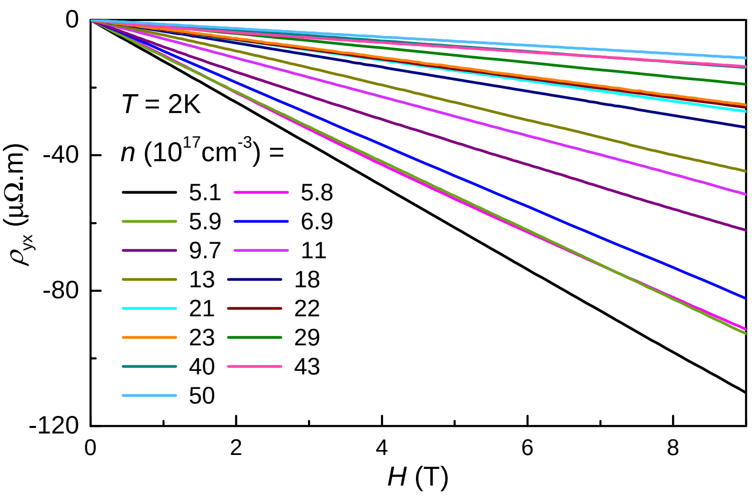

Supplementary Figure 3: **Hall resistivity (ρ_{yx}) as a function of magnetic field (H) at 2K for different samples in this study**. $\rho_{yx}(H)$ is linear as expected from the single-electronic-band structure. The negative slope indicates samples are electron doped. Relevant transport parameters are listed in Supplementary Table 2. The low-T Hall electron mobility is over 35000 $cm^2.V^{-1}.s^{-1}$, along with a specimen at $n \approx 5 \times 10^{18}$ cm^{-3} exhibits mobility around 320000 $cm^2.V^{-1}.s^{-1}$, belong to the best value ever reported [1, 2].

Supplementary Table 2: **Transport parameters for Bi_2O_2Se samples in this study.** n is Hall carrier concentration; A is the prefactor of T^2 resistivity; ρ_0 is the residual resistivity; RRR is the room-temperature residual resistivity ratio; μ_{2K} is the Hall electron mobility measured at 2K.

Sample	n cm^{-3}	A $\mu\Omega.K^{-2}$	ρ_0 $\mu\Omega$.cm	RRR	μ_{2K} $cm^2.V^{-1}.s^{-1}$
1	5.1×10^{17}	0.252	333	233	36875
2	5.8×10^{17}	0.199	262	178	41402
3	5.9×10^{17}	0.261	208	281	51107
4	6.9×10^{17}	0.0933	73.9	423	123243
5	9.7×10^{17}	0.0884	74.9	267	86363
6	1.1×10^{18}	0.135	163	200	34916
7	1.3×10^{18}	0.0752	74.6	251	63021
8	1.8×10^{18}	0.0433	38.2	261	91322
9	2.1×10^{18}	0.0453	50.3	191	60073
10	2.2×10^{18}	0.0522	25.3	392	114494
11	2.3×10^{18}	0.0594	69	203	40235
12	2.9×10^{18}	0.0324	11.9	551	181528
13	4×10^{18}	0.025	10.5	317	148952
14	4.3×10^{18}	0.0391	21.2	423	69220
15	5×10^{18}	0.00802	3.87	346	324348
16	1.1×10^{19a}	0.00336	3.32	452	224000

a. This specimen is from Ref. [2]

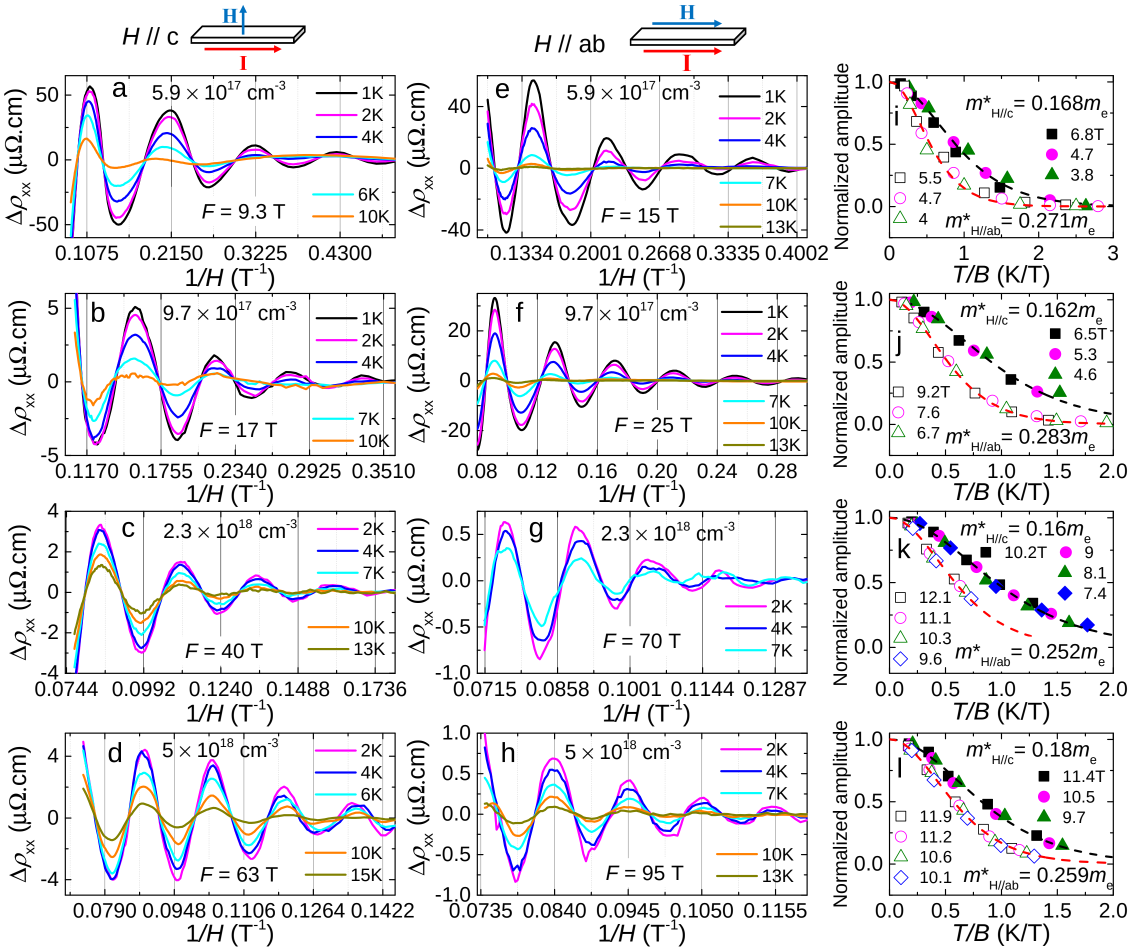

Supplementary Figure 4: **Shubnikov-de Haas effect for other Bi_2O_2Se samples with various carrier concentrations (n). a-d** SdH effect for four samples with H along c-axis. **e-h** SdH effect for the same samples with H along ab-plane. **i-l** Temperature dependent oscillation amplitudes. The effective mass (m^*) is extracted by fitting with L-K formula (The dashed lines). The oscillation frequency (F) grows monotonically with increasing n leaving the mass less changed. Relevant data are summarized in Fig. 3e and Supplementary Table 3.

Supplementary Table 3: **Data from SdH effect for selected samples of Bi_2O_2Se.** n is the Hall carrier concentration; $F_{H\|c}$ is the in-plane oscillation frequency; $m^*_{H\|c}$ is the in-plane effective mass; $F_{H\|ab}$ is the out-of-plane frequency; $m^*_{H\|ab}$ is the out-of-plane effective mass; α is the Fermi surface anisotropy; n_{SdH} is the carrier concentration extracted from oscillations.

n cm^{-3}	$F_{H\|c}$ T	$m^*_{H\|c}$ m_e	$F_{H\|ab}$ T	$m^*_{H\|ab}$ m_e	α	n_{SdH} cm^{-3}
5.9×10^{17}	9.3	0.168	15	0.271	1.61	2.6×10^{17}
9.7×10^{17}	17	0.162	25	0.283	1.47	5.8×10^{17}
2.3×10^{18}	40	0.16	70	0.252	1.75	2.5×10^{18}
4.3×10^{18}	51	0.17	93	0.254	1.82	3.8×10^{17}
5×10^{18}	63	0.18	95	0.259	1.51	4.3×10^{17}

Supplementary Table 4: **Data for samples in Fig. 4b.** The Fermi temperature (E_F/k_B) is calculated from the slope of thermopower (S/T) at low temperatures through $|S/T| = \frac{\pi^2}{2}\frac{k_B}{e}\frac{k_B}{E_F}$. A is the prefactor of T^2 resistivity.

Samples	S/T<br>μV.K^{-2}	E_F/k_B<br>K	A<br>$\mu\Omega$.cm.K^{-2}
$CuRh_{0.9}Mg_{0.1}O_2$ [4]	0.43	991	5.87E-3
$CuRh_{0.94}Mg_{0.06}O_2$ [4]	0.72	592	0.0114
URu_2Si_2 [5, 6]	3	141	0.126
$Ca_3Co_4O_9$ [7]	2.6	164	0.036

Supplementary Table 5: **Data for conventional metals and heavy Fermions in Fig. 4b from [8].** γ is the electronic specific heat coefficient. E_F/k_B is deduced from γ by taking n_e = 1 e per f.u..

Samples	γ<br>mJ.mol^{-1}.K^{-2}	E_F/k_B<br>K	A<br>$\mu\Omega$.cm.K^{-2}
Os	2.5	16404	2.2E-6
Re	3.0	13670	3.8E-6
Fe	4.9	8369	1.3E-5
Pt	6.4	6408	1.4E-5 to 2E-5
Ni	7.1	5776	9.5E-6 to 2.6E-5
Pd	9.3	4410	3.3E-5
CeB_6	250	164	0.83
$YbRh_2Si_2$ (6T)	300	137	1
$YbRh_2Si_2$	1700	24	22

Supplementary Table 6: **Data for correlated metals, semimetals and doped semiconductors in Fig. 4b.** E_F/k_B is measured from quantum oscillations. α, β et al. mark different Fermi pockets. (100) and (110) mark magnetic field orientations along different crystal axis. h and e mark hole and electron pockets respectively.

Samples	Bands	E_F/k_B<br>K	A<br>$\mu\Omega$.cm.K^{-2}
UPt_3[8]	γ<br>ω	24<br>87	1.55
$CeRu_2Si_2$[8]	110 α<br>100 β	53<br>845	0.94
Sr_2RuO_4[8]	α-h<br>β-e	1240<br>2450	6.1E-3
YBCO(p=0.11)[8]		410	8.5E-3
Bi[8]	h<br>e	127.6<br>313.2	0.012
$Bi_{0.96}Sb_{0.04}$[8]	h<br>e	34.8<br>140.4	0.033
Graphite[8]	e<br>h	278<br>336	6.4E-3
WTe_2[9]		232 to 464	4.8E-3
Cd_3As_2[10]		1777	6.25E-3
$ZrTe_5$[11]		128 (359)	0.1 (0.036)
$EuTiO_3$[12]		282	0.086

Supplementary Discussion

Electron-soft TO phonon scattering. The electron-soft transverse optic phonon (TO) scattering mechanism is proposed by Epifanov[3]. In this picture, the electron-ferroelectric soft phonon coupling leads to an electron relaxation time (τ) proportional to $\frac{1}{T^2}$. The equation is complex:

$$\tau(E) = \frac{128\pi^3\hbar^3\delta^2}{g^2 m^* k_{\mathrm{B}}^2 \varphi(l)} \frac{1}{T^2} \quad (1)$$

where $\hbar$ is the reduced Planck constant, k_{B} is Boltzmann constant, m^* is the effective mass of electrons, g is an electron-phonon interaction constant, δ is a constant reflecting the wave number dependence of permittivity $\varepsilon(q) = [\varepsilon_0^{-1} + \delta q^2]^{-1}$, ε_0 is the static permittivity, q is the phonon wave number and $\varphi(l)$ is a complex power-law formula with $l = 2mE\varepsilon_0\delta\hbar^{-2}$ and E the kinetic energy.

Now, since $SrTiO_3$ is quantum paraelectric, such a scenario cannot be excluded there. On the other hand, Bi_2O_2Se is not a quantum paraelectric and there is no reason to suspect the relevance of this scenario.

---

[1] Chen, C. *et al.* Electronic structures and unusually robust bandgap in an ultrahigh-mobility layered oxide semiconductor, Bi_2O_2Se. *Sci. Adv.* **4**, eaat8355 (2018).

[2] Lv, Y. Y. *et al.* Electron-electron scattering dominated electrical and magnetotransport properties in the quasi-two-dimensional Fermi liquid single-crystal Bi_2O_2Se. *Phys. Rev B* **99**, 195143 (2019).

[3] Epifanov, Y. N., Levanyuk, A. P. & Levanyuk, G. M. Interaction of carriers with TO-phonons and electrical conductivity of ferroelectrics. *Ferroelectrics* **35**, 199-202 (1981).
24, 1049-1054 (1960).

[4] Kurita, K., Sakabayashi, H. & Okazaki, R. Correlation in transport coefficients of hole-doped $CuRhO_2$ single crystals. *Phys. Rev. B* **99**, 115103 (2019).

[5] Palstra, T. T. M.,Menovsky, A. A. & Mydosh, J. A. Anisotropic electrical resistivity of the magnetic heavy-fermion superconductor URu_2Si_2. *Phys. Rev. B* **33**, 6527-6530(R) (1986).

[6] Sakurai, J., Hasegawa, K., Menovsky, A. A. & Schweizer, J. Thermoelectric power on single crystals of URu_2Si_2. *Solid State Commun* **97**, 689-691 (1988).

[7] Saito, K. & Okazaki, R. Electron correlation effect in the thermoelectric oxide $Ca_{3-x}Sr_xCo_4O_9$ single crystals. *Jpn. J. Appl. Phys.* **56**, 043201 (2017).

[8] Lin, X., Fauqué, B. & Behnia, K. Scalable T^2 resistivity in a small single-component Fermi surface. *Science* **349**, 945 (2015).

[9] Zhu, Z. W. *et al.* Quantum oscillations, thermoelectric coefficients, and the Fermi surface of Semimetallic WTe_2. *Phys. Rev. Lett.* **114**, 176601 (2015).

[10] He, L. P. *et al.* Quantum Transport Evidence for the Three-Dimensional Dirac Semimetal Phase in Cd_3As_2. *Phys. Rev. Lett.* **113**, 246402 (2014).

[11] Martino, E. *et al.* Two-dimensional conical dispersion in $ZrTe_5$ evidenced by optical spectroscopy. *Phys. Rev. Lett.* **122**, 217402 (2019).

[12] Maruhashi, K. *et al.* Anisotropic quantum transport through a single spin channel in the magnetic semiconductor $EuTiO_3$. *Adv. Mater.* **32**, 1908315 (2020).
